# What occupational therapists’ say about their competencies’ enactment, maintenance and development in practice? A two-phase mixed methods study

**DOI:** 10.1186/s12909-020-02087-4

**Published:** 2020-06-15

**Authors:** Annie Rochette, Martine Brousseau, Brigitte Vachon, Cynthia Engels, Fatima Amari, Aliki Thomas

**Affiliations:** 1 Occupational Therapy Program, School of Rehabilitation, University of Montreal, C.P. 6128, Succursale Centre-Ville, Pavillon Parc, Montréal, Québec H3C 3J7 Canada; 2 Centre for Interdisciplinary Research in Rehabilitation in greater Montreal (CRIR), Montréal, Canada; 3 Occupational Therapy Department, Université du Québec à Trois-Rivières, Trois-Rivières, Canada; 4 Centre de recherche de l’Institut universitaire en santé mentale de Montréal, Montréal, Canada; 5 Univ Paris Est Creteil, INSERM, IMRB, Equipe CEpiA, F-94010 Creteil, France; 6 Univ Paris Est Creteil, UFR de santé, Institut de formation en ergothérapie, F-94010 Creteil, France; 7 School of Physical and Occupational Therapy, McGill University, Montréal, Canada; 8 Institute of Health Sciences Education, McGill University, Montréal, Canada

**Keywords:** Occupational therapists, Professional competence, Professional role, Surveys and questionnaires, Focus groups, Perception, Organizations

## Abstract

**Background:**

Understanding how professional competencies are actually enacted in clinical practice can help university programs better prepare their graduates. The study aimed to describe 1) the perceived competency level of occupational therapists holding an entry-to-practice master’s degree 2) the factors perceived as impacting the enactment of competencies; and 3) the strategies used to maintain and further develop level of competency in the seven practice roles: expert in enabling occupation, communicator, collaborator, practice manager, change agent, scholarly practitioner and professional.

**Methods:**

Descriptive two-phase mixed methods sequential design. The quantitative phase consisted of an online survey sent to all occupational therapists holding an entry-to-practice master’s degree in Quebec, Canada (*n* = 1196), followed by focus group discussions with a subset of participants. Analysis used descriptive statistics and the Framework Approach for content analysis of focus group data. Competencies were theoretically anchored into the Profile of Occupational Therapist Practice in Canada (an equivalent to CanMEDS framework) and we used the Theoretical Domain Framework to collect and describe perceived factors and strategies.

**Results:**

Response rate to Phase 1 was 26.5% (*n* = 317/1196). The communicator, collaborator and professional roles were perceived as highly solicited in practice, valued and were rated more frequently at a higher competency level as compared to the other four roles (expert, manager, change agent and scholar roles). Focus group participants (*n* = 16) mentioned that both individual and organisational factors influence enactment of competencies. Consulting colleagues was the preferred strategy to support the scholar role, often described as foundational for the development of expertise.

**Conclusion:**

This descriptive study provides valuable information as to how the seven roles are enacted in practice. Though the scholar role is highly valued by clinicians and organizations, insufficient time is allotted to searching for evidence and reflection in practice. Strategies emerging from the results are mainly directed toward this role with an emphasis on using of peers as a source of evidence. Future studies could explore how contextual factors influence the enactment of competencies across different professions as well as how these evolve over time.

## Background

Assessment of competence and maintenance of standards in many health care professions, including occupational therapy, have been growing areas of discussion and interest since the early 2000’s in many countries [1]. An upgrade of the minimum standards for entry level practice to the graduate level was deemed necessary given the proliferation of new knowledge emerging from research, the increased complexity of practice environments, the rise in clients presenting with multiple diagnoses and the recurring reorganization of health care and changing structures [2]. A consultation of close to 2200 members of the Canadian Association of Occupational Therapists revealed that respondents agreed that the knowledge and competence levels once deemed only required for ‘advanced practice’ in 1996 had become essential for all practising occupational therapists in 2002 [3]. Therefore, as of 2008, an entry-to-practice master [4] was the mandatory minimum requirement to practise occupational therapy in Canada. The aim of the upgrade in an entry-to-practice level education was to enhance graduates’ competency level in the seven roles of the Profile of practice of occupational therapists in Canada [4] used for accreditation of university programs, an equivalent to CanMEDS [5] for the occupational therapy profession.

The seven roles (each comprised of key-competencies further divided into several enabling competencies) included in the Profile of Occupational Therapist Practice in Canada (hereafter being referred to as the Profile), first published in 2007 [4], were identified and validated using a five-phase process. The profile was inspired by CanMEDS framework developed for medicine but adapted for the occupational therapy profession [6, 7]. The central role in occupational therapy is the expert in enabling occupation and is surrounded by six transversal roles: communicator (e.g. engaging in effective dialogue and written documentation) collaborator (e.g. working in teams and managing conflict), practice manager (e.g. prioritizing duties and supervision of trainees), change agent (e.g. advocating for occupation), scholarly practitioner (e.g. using of evidence-based practice and reflexive practice) and professional (e.g. committed to ethical practice and high personal standards of behaviour) [4].

Although Lal, Klein & Brown [3] reinforced the importance of studying the competence level of occupational therapists with master’s entry-to-practice educational preparation, only a few articles on the topic exist. Two studies compared the perceived competence level of occupational therapists holding a baccalaureate versus a master’s. One study dating back to over 30 years, found that those with a master’s degree reported greater confidence in their competence as compared to those with a baccalaureate [8]. In another study, while both groups searched for information for similar reasons, those holding a master’s frequently used scientific databases to search information, whereas those with a baccalaureate often consulted colleagues for similar information [9]. Another thirteen articles, one being more than 30 years old, focused primarily on one of the seven roles with the scholar role being the most studied (8/13 articles) [9–16], followed by the role of expert in enabling occupation [17, 18], collaborator [19, 20] and only one focusing on the professional role [21]. Enactment of these competencies does not solely rely on occupational therapists’ initial training and understanding of personal and contextual factors that facilitate or hinder their use, maintenance and development in authentic practice settings is urgently needed.

## Objective

The overarching aim of this study was to describe the ways in which occupational therapists (OTs) with an entry-to-practice master’s degree perceived their enactment of the seven roles [4] in actual practice. More precisely, we aimed to describe:

1. clinicians’ perceived competency level for the seven roles of the Profile [4];
2. the individual and organizational factors (facilitators and barriers) perceived as impacting clinicians’ ability to enact key competencies relating to the seven roles in their daily practice and;
3. the strategies used by OT clinicians with an entry-to-practice master’s degree to maintain and further develop their competency level for the seven roles.

This study endorses the definition of competence at an individual level as the effective combination and mobilization of internal (i.e. skills, attitudes, knowledge) and external resources (e.g. working environment, roles and responsibilities, physical and organizational demands) in a set of situations and contexts [22].

## Methods

We used a two-phase mixed methods sequential design [23, 24]. Phase 1 consisted of an online survey (objective 1) and phase 2 involved qualitative focus group discussions (objectives 2 and 3) with the aim to deepen our understanding of phase 1 results. Ethics approval was obtained from the Health Research Ethics Board of the University of the first author. Participants provided written consent to participate to phase 1 and further verbal consent to participate to phase 2 as focus groups were held online.

### Participants

The study was conducted in Quebec, Canada. All occupational therapists holding an entry-to-practice master’s degree, who are members of the *Ordre des ergothérapeutes du Québec*, the Provincial regulatory organization, and who consented to be contacted for research purposes when submitting their annual registration renewal, were eligible to participate in this study (*n* = 1196) and were therefore contacted through e-mail. There were no exclusion criteria.

### Data collection for phase 1

The online survey was developed simultaneously in French and English (given the bilingual demographic of this large Canadian province) using a four-step process adapted from Devellis’ 8-step process of scale development [25]: 1) a list of indicators for each of the seven roles of the Profile [4] was developed based on literature; 2) indicators were added to this list to ensure all seven roles were covered; these were framed as questions by the research team using a constant iterative process moving between the literature and the research team discussions until consensus was reached; 3) this beta version of the survey was improved for its content using focus group discussion [26] with academic experts (n = two groups of 4–5 individuals) as well as individual interviews (*n* = 3) with managers; 4) this first version was then pre-tested online (*n* = 60). This process resulted in a reduction in the number of questions (72 questions initially reduced to 40 questions) to ensure it could be completed in less than 10 min. The final survey was composed of 46 questions including sociodemographic characteristics.

### Data collection for phase 2

This second phase targeted objectives 2 and 3 of the study. Participants from phase 1 (online survey) were invited to provide their contact information if they agreed to be contacted (*n* = 103/317; 32.5%) for phase 2 (focus group discussion). For this second phase, purposeful sampling was used to include, occupational therapists working with a variety of clientele of all ages including physical and mental health as well as public and private practice settings. The focus group protocol included open-ended questions targeting three main areas: 1) overall perceived competency level (i.e. which role was perceived as highest versus lowest competency level); 2) individual and organizational factors [27] perceived as impacting one’s ability to enact key competencies in practice; and 3) strategies used in practice to maintain and further develop competency level. A list of probes (e.g. Which (roles) do you consider better or worse for you or your colleagues and can you give a concrete example? What about ‘expert in enabling occupation’? In other words, we asked them to provide examples of situations in which the different roles were perceived as being enacted and to further comment on how these were either easy or challenging) for each of these three areas was used as needed to foster discussion. We used a semi-inductive approach as the probes used to collect information about individual and organizational factors were anchored in the Theoretical Domains Framework [27]. All focus groups were conducted in French (no first English-language speakers volunteered for phase 2), recorded and transcribed verbatim. The quotes used in the manuscript were translated using www.deepl.com and further checked for accuracy by bilingual team members.

### Data analysis

We used descriptive statistics (mostly frequency and percentage) to analyze the survey data and Pearson Chi square statistics or exact Fisher’s test (two-sided test) to explore associations between perceived competence level on the seven roles and specific variables (e.g. time since graduation, taking on a preceptor role, working in private/public setting and clientele being physical/mental health). Analysis was done using IBM SPSS statistics Version 25. Focus group data (transcribed verbatim) were analyzed using the Framework approach [28]. This thematic approach allows for making explicit links within the different steps of the analysis; an iterative process is used to move between raw data, codes and emerging themes. We used the seven roles of the Profile [4] and the components of the Theoretical Domains Framework [27] as an initial coding grid to which open coding was added. The Theoretical Domains Framework was developed to investigate factors potentially influencing specific behaviours change in practice and its refined version contains 14 domains (e.g. knowledge, beliefs about capabilities or social influences) [27]. Coding and results were discussed in a team meeting until consensus was reached. Trustworthiness [29] was established through the following processes: Authenticity was addressed by using probes during focus groups and inviting participants to provide concrete examples corresponding to their specific contexts. Confirmability was achieved by having a different co-facilitator from the research team and an observer taking notes in a log book. This was further promoted in data analysis by using theories (Profile and Theoretical Domains Framework) as a preliminary coding grid. The log also ensured dependability, that is, stability of results in similar conditions, by noting any changes made in the research process. Finally, to ensure credibility, available data from all six focus groups were included into the data analysis and all participants were invited to freely express their views using the ‘turn and talk’ procedure. We also used an iterative process where emerging themes from one focus group was further explored with subsequent ones (e.g. *Previous participants told us x, what do you think, is it the same for you?*) Credibility was also achieved using triangulation, that is, different sources of information such as the survey (phase 1), log and post focus groups synthesis were used throughout the analytical process.

## Results

### Sample characteristics

Characteristics of online survey respondents (Phase 1) are presented in Table 1. The response rate was 26.5% (*n* = 317/1196). Most respondents held a clinician role (*n* = 308/317; 97.2%) whereas 17% (54/317) held multiple roles. Three quarters of participants (237/316) were working full time at the time of the survey and 86.2% (257/298) worked for one employer. More than half (161/265; 60.8%) held a permanent position. A ratio of 2:1 clinicians worked with adults and older adults as compared to youth and young children and in physical health as compared to mental health.

**Table 1:** Participant Socio-demographics (*n* = 317)

	*N* (%)	*M*(SD)		*N* (%)
Age, years (*N* = 317)		28.4 (3.9)	Practice Location (N = 317)^a^	
*24–28*	202 (63.7)		*Hospital*	51
*29–33*	87 (27.4)		*Rehabilitation Centre*	66
*34–38*	19 (6.0)		*Local Community Service Center*	71
*39–43*	7 (2.2)		*Long-term care facility*	33
*44 and over*	2 (0.7)		*Private clinic*	100
Years of Graduation (*N* = 314)		3.6 (1.9)	*Own practice*	16
*2016 (Less than one year)*	61 (19.2)		*Consultant*	6
*2014–15*	120 (37.9)		*School environment*	16
*2012–13*	97 (30.6)		*University*	14
*2007–2011 (More than 5 years)*	39 (12.3)		*Research centre*	11
			*Other*	10
Gender (N = 317)			Professional Activity (*N* = 316)	
*Male*	14 (4.4)		*Full time*	237 (75)
*Female*	301 (95.0)		*Part time between 50 and 99%*	63 (20)
*I prefer not to respond*	2 (0.6)		*Part time < 50%*	16 (5)
University (N = 317)			Professional Activity (*N* = 298)	
*McGill University*	42 (13.2)		*1 employer*	257 (86.2)
*Université Laval*	52 (16.4)		*2 or more employers*	41 (13.8)
*Université de Montréal*	132 (41.6)		Professional Activity (*N* = 265)	
*Université du Québec à Trois Rivières*	41 (12.9)		*Permanent job*	161 (60.8)
*Université de Sherbrooke*	45 (14.2)		*Replacement*	104 (39.2)
*Other*	5 (1.6)			
Work Situation (N = 317)			Supervisor’s Discipline (*N* = 315)	
*Employed*	292 (92.2)		*Occupational Therapist*	126 (40)
*Looking for a job*	3 (0.9)		*Physiotherapist*	45 (14.3)
*Studying*	4 (1.3)		*Social worker*	36 (11.4)
*Employed and looking for Job*	3 (0.9)		*Nurse*	27 (8.6)
*Employed and studying*	6 (1.9)		*Doctor*	4 (1.3)
*Parental Leave*	6 (1.9)		*I own my clinic*	3 (1.0)
*Other*	3 (0.9)		*I work on private practice*	2 (0.6)
Position (N = 317)^a^			*More than one answer*	7 (2.2)
*Clinician*	308		*I do not know*	3 (1.0)
*Student*	20		*Other*	62 (19.7)
*Clinician Coordinator*	12			
*Researcher*	11		Clientele (N = 316)^a^	
*Lecturer in an OT program*	10		*Older adults (65 yr +)*	174
*Consultant*	8		*Adults (18–64 yrs)*	217
*Manager*	5		*Youth (5–16 yrs)*	103
*Special project manager*	3		*Young children (0–4 yrs)*	75
*Lecturer in a program other than OT*	1		*Physical Health*	202
*Continuing education instructor*	1		*Mental Health*	103
*Multiple Roles*	54 (17.0)		*Other*	3
*Other*	4		*Not Applicable*	1

N varies because of missing data, *M(SD)* Mean (Standard Deviation), ^a^ Could provide multiple answers

The phase 2 (focus groups) sample was comprised of 16 occupational therapists from 3 out of 5 Quebec university OT training programs. The majority (12/16) was working full time, half (8/16) held a permanent position and they represented a variety of work settings: 4/16 worked in a hospital, 6/16 in a rehabilitation centre, 1/16 in a local community services centre, 1/16 in a long-term care institution, 6/16 work in a private clinic and 1/16 in a school (with 3/16 having more than one employer).

### Objective 1: overall perceived competency level

When asked how they would rate their competency level for each of the seven roles, the majority rated it as high or very high for the communicator, collaborator and professional roles. No respondent rated these as inadequate (see Table 2). The other four roles were perceived as adequate or better for half of respondents. Three of the seven roles were rated as borderline or inadequate (i.e. below the expected level) (proportion, n varies because of missing data) for 10.2% (31/304) of respondents for the practice manager role, for 17.2% (52/303) for the scholarly practitioner role and for 21.0% (63/303) for the change agent role.

**Table 2:** Global perceived level of competency and percentage of time spent on activities and approaches (highest proportion is bolded)

*Currently, how would you rate your competency level, in each of the seven roles, as defined by the CAOT?*						
Roles presented in Profile’s order	Inadequate *n* (%)	Borderline *n* (%)	Adequate n (%)	High *n* (%)	Very high *n* (%)	I don’t know *n* (%)
Expert in Enabling Occupation (*N* = 304)	1 (0.3)	9 (3.0)	**152 (50.0)**	103 (33.9)	39 (12.8)	0
Communicator (*N* = 303)	0	9 (3.0)	121 (39.9)	**124 (40.9)**	49 (16.2)	0
Collaborator (N = 303)	0	5 (1.7)	63 (20.8)	**161 (53.1)**	72 (23.8)	2 (0.7)
Practice Manager (N = 304)	1 (0.3)	30 (9.9)	**142 (46.7)**	101 (33.2)	29 (9.5)	1 (0.3)
Change Agent (N = 303)	3 (1.0)	60 (19.8)	**155 (51.2)**	65 (21.5)	18 (5.9)	2 (0.7)
Scholarly Practitioner (N = 303)	2 (0.7)	50 (16.5)	**158 (52.1)**	79 (26.1)	13 (4.3)	1 (0.3)
Professional (N = 304)	0	1 (0.3)	74 (24.3)	**149 (49.0)**	80 (26.3)	0
*On average, what percentage of your time per week is spent on the following activities?*						
		0–19% n (%)	20–39% n (%)	40–59% n (%)	60–79% n (%)	80–100% n (%)
Direct time with clients (*n* = 302)		19 (6.3)	**109 (36.1)**	99 (32.8)	60 (19.9)	15 (5.0)
Preparing for sessions with clients (*n* = 300)		**276 (92.0)**	24 (8.0)	0	0	0
Formal/informal meetings or discussions (*n* = 299)		**263 (88.0)**	33 (11.1)	3 (1.0)	0	0
Writing case notes (clients’ documentation) (*n* = 301)		82 (27.2)	**168 (55.8)**	51 (16.9)	0	0
Reading or looking for evidence-based data (*n* = 289)		**282 (97.6)**	5 (1.7)	0	2 (0.7)	0
Administrative tasks (*n* = 287)		**271(94.4)**	14 (4.9)	2 (0.7)	0	0
*What percentage of your direct time with clients do you spend on the following approaches per year?*						
		0–19% n (%)	20–39% n (%)	40–59% n (%)	60–79% n (%)	80–100% n (%)
Individual (*n* = 293)		18 (6.2)	27 (9.2)	23 (7.8)	22 (7.5)	**203 (69.3)**
Individual with more than one client at a time (*n* = 208)		**142 (68.3)**	14 (6.7)	14 (6.7)	16 (7.7)	22 (10.6)
In group (*n* = 210)		**170 (81.0)**	25 (11.9)	10 (4.7)	5 (2.4)	0 (0)
Populational (*n* = 168)^a^		**158 (94.0)**	6 (3.6)	1 (0.6)	2 (1.2)	1 (0.6)

^a^Response rate is probably explained by respondents perceiving this context of practice as not applicable to their situation

Phase 2 participants provided more insight with those results: *I would agree with the survey results. Manager and Change agent I find so much that it is what requires the most energy and is often the least rewarding to spend energy* [Participant (P)3]. *In fact, when you named the strongest, and the ones who came out as the weakest, it speaks to me, they are classified like that...* [P11].

The scholar role, although perceived as valued by employers was referred to as a role that was being enacted on personal time: *Well, if I think about my practice setting, what is at the level of research, knowledge sharing, it’s valued, but it will always be done outside of working hours. During lunch hours... So yes, it is welcomed with open arms, but it will always be on a voluntary basis.* [P1]. The scholar role was reflected in practice as the opportunity to access continuing professional development activities with a challenge to apply new knowledge in practice: *I was thinking, Scholar.... I tend to get a lot of training because I like to learn..., but I still find that sometimes it’s difficult to apply what you learn in the clinic on certain aspects, because you don’t have enough experience. We would like to try to integrate a new service, but we don’t know where to start.* [P4].

Practice manager role was especially perceived as challenging. Focus group participants expressed difficulties in effectively managing waiting list and setting priorities on a daily basis: *As for Manager, I experience it in my practice because I am the one who has to manage my waiting list for clinical cases. It’s definitely something I didn’t feel very comfortable doing at first*. [P6]. *I would just like to add that I agree with that for the manager’s role point. Because I too have to manage my waiting list and I too was not comfortable with that and I talked to my manager about it to help me prioritize the list a little bit*. [P7]. *I would also say practice manager. That I think was one of the competencies that was the lowest. I would say that I am in a context of intensive rehabilitation, so it is for sure that prioritizing, prioritizing tasks, trying to get there, trying to get there most effectively, is more difficult... And I think that in practice manager there is also the point that it is trying to balance work and life, say, outside activities, those kinds of things...* [P2].

### A portrait of the seven roles in practice

#### Expert in enabling occupation: the central role

The role of expert in enabling occupation is the central role. The great majority of respondents mentioned practising on a one-on-one, individual basis with their clients. Practising on an individual basis but with more than one client at a time and in groups are used in combination although no respondent mentioned practising only in a group mode (see Table 2).

The main reasons for referral varied and mentioned a specific diagnostic (clientele), targeted the environment (mainly for adaptation) or function (in general or specific). Thirty-one/317 (9.8%) respondents did not mention any models used in practice whereas nearly 15.1% (48/317) mentioned two or more. The most frequently used model was the Canadian Model of Occupational Performance and Engagement - CMOP-E [30] in a proportion of 67.8% (215/317) followed by the Disability Creation Process [31] (11.7%; 37/317) and the Model of Human Occupation-MOHO [32] (7.3%; 23/317). The biomechanical approach [33] was used most frequently by 20.8% (66/317) of respondents, followed by an adaptation/rehabilitation approach (19.6%; 62/317) and a sensory integration approach (15.1%; 48/317). Respondents listed a total of 73 different standardized assessment tools used in practice. However, 64/317 (20.2%) did not mention any, and 103/317 (32.5%) listed five (maximum survey space allowed). The two most frequent interventions targeted by respondents were skill development/training and environmental modifications; whereas the least frequent was information/education and support to relatives.

#### The six supporting roles: an overview

An overview of reported practice in the six supporting roles is presented in Table 3. These roles are presented in order from highest perceived level of competency to lowest (when adding proportion of respondents who rated these as high or very high- See Table 2): Collaborator (76.9%), Communicator (57.1%), Professional (45.3%), Practice manager (42.7%), Scholarly practitioner (30.4%) and finally, Change Agent (27.4%).

**Table 3:** Overview of practice in the six supporting roles presented in order of highest to lowest proportion of perceived competency level *Frequency of exposition to situations* or *level of agreement with quotes*; most frequent is bolded (n = 304 and varies because of missing data)

**Collaborator**	This role is perceived as being highly solicited and valued: “*We collaborate with really, really many people*”. [P11]; “*Collaborator is quite easy. Even our institution requires us to make many interdisciplinary intervention plans, so teamwork is very highly valued*.” [P12].						
*Who are the partners you typically collaborate with in your practice? (check all that apply)* top 4 are presented (*n* = 303)	OT 263 (86.8%)	PT 232 (76.6%)	Clients’ relatives 228 (75.2%)	Doctors/specialists 225 (74.3%)	Mean = 9.8 ± 3.8 Median = 10; Mode = 12 Range = 2 to 24 different collaborators		
		Strongly disagree n (%)	Disagree n (%)	Neutral n (%)	Agree n (%)	Strongly agree n (%)	Not applicable n (%)
*I can manage conflicts in my team* (n = 304)		0 (0)	13 (4.3)	90 (29.6)	**139 (45.7)**	24 (7.9)	38 (12.5)
*I can manage conflicts with professionals outside my team*(n = 304)		1 (0.3)	15 (4.9)	95 (31.3)	**119 (39.1)**	11 (3.6)	63 (20.7)
**Communicator**	Respondents (n = 301) estimated spending on average 24.9 ± 11.8% of their time in writing charts (see Table 2) which is the second most important activity after spending direct time with clients (average of 43.4% of week time ± 19.1%; *n* = 302). A participant in phase 2 corroborated this: “*I find that* [writing charts] *takes a lot of time in my practice. I feel like I can’t see the clientele as much as... I should see people more than that in my weeks, but I can’t because I have too much writing to do*” [P12]. A colleague added “*And I really agree with my colleagues at the communicator level, as far as oral communication is concerned, it’s still going well, it’s really written communication that poses the challenge. Indeed, the writing time is enormous, and I find that I am falling behind like that cannot be done. From there comes the role of manager, to know how to organize my week well and also to know how to be succinct in my charts’ writing*” [P11].						
		Never n (%)	Rarely n (%)	Occasionally n (%)	Often n (%)	Very Often n (%)	Everyday n (%)
*In my clinical environment, I am exposed to complex (multidimensional) situations* (*n* = 303)		0 (0)	23 (7.6)	**113 (37.3)**	0 (0)	107 (35.3)	60 (19.8)
**Professional**	This role was not perceived as challenging. As P12 stated “*At the professional level there is no problem at all.*”						
		Strongly disagree n(%)	Disagree n(%)	Neutral n(%)	Agree n(%)	Strongly agree n(%)	Not applicable n(%)
*I manage to deal effectively with ethical situations in my practice* (*n* = 304)		0 (0)	10 (3.3)	80 (26.3)	**187 (61.5)**	23 (7.6)	4 (1.3)
**Manager**	This role is perceived as especially challenging.						
		Strongly disagree n (%)	Disagree n (%)	Neutral n (%)	Agree n (%)	Strongly agree n (%)	Not applicable n (%)
*I manage my time effectively in my various professional activities* (n = 304)		2 (0.70)	68 (22.4)	63 (20.7)	**137 (45.1)**	34 (11.2)	0 (0)
*Considering the scarcity of resources, I manage to find solutions to achieve the objectives set for my clients* (n = 303)		0 (0)	27 (8.3)	65 (21.5)	**179 (59.1)**	25 (8.3)	7 (2.3)
*I supervise non-occupational therapy staff effectively* (n = 304)		3 (1.0)	12 (3.9)	54 (17.8)	**110 (36.2)**	18 (5.9)	107 (35.2)
*I play a leadership role in the team* (n = 304)		2 (0.70)	42 (13.8)	**124 (40.8)**	102 (33.6)	27 (8.9)	7 (2.3)
*How much time do you spend on committees/working group for the institution where you work?* (n = 302)		Not involved 110 (36.4%)	Few hours/year 81 (26.8%)	Few hours/month 57 (18.9%)		Few hours/week 10 (3.3%)	Not applicable 44 (14.6%)
*I supervise trainees/interns* (*n* = 246 eligible as having more than one year since graduation)		No 143/246 (58.1%)			Yes 103/246 (41.9%)		
**Scholarly practitioner**	While the vast majority of respondents (294/303; 97.1%) agree or strongly agree with the statement *I understand the term evidence-based practice*, the proportion decreases to nearly half (160/303; 52.8%) for those who agree or strongly agree with *being aware of evidence-based practice in my profession*						
		Strongly disagree n (%)	Disagree n (%)	Neutral n (%)	Agree n (%)	Strongly agree n (%)	Not applicable n (%)
*I understand the term evidence-based practice* (n = 303)		2 (0.7)	0 (0)	7 (2.3)	119 (39.3)	**175 (57.8)**	0 (0)
*I am aware of evidence-based practice in my profession* (n = 303)		3 (1.0)	44 (14.5)	96 (31.7)	**129 (42.6)**	31 (10.2)	0 (0)
*I try to develop skills in accessing, acquiring and appraising evidence relevant to my area of practice* (n = 303)		5 (1.7)	15 (5.0)	51 (16.8)	**172 (56.8)**	59 (19.5)	1 (0.3)
*I read relevant literature* (n = 303)		3 (1.0)	30 (9.9)	73 (24.1)	**160 (52.8)**	37 (12.2)	0 (0)
*Evidence-based practice helps me to make decisions about clients in my work* (n = 303)		3 (1.0)	11 (3.6)	90 (29.7)	**160 (52.8)**	38 (12.5)	1 (0.3)
**Change agent**	is also perceived especially as challenging. It could be defined as a second degree of the expert in enabling occupation and for some, remains unclear: “*Change agent even when we were doing the portfolio at university, we would say that it was always the one* [role] *we didn’t really know what to write about in there and I would say that it is even going to practise... change agent we try to do more in everyday life, to teach to those in charge of resource, to try to educate... it’s more small changes like that that that I find we are able to make, by our knowledge and the way we behave that we can influence colleagues and partners. But change agent remains a challenge, to put bigger things in place, it’s not easy*” [P11].						
		Strongly disagree n (%)	Disagree n (%)	Neutral n (%)	Agree n (%)	Strongly agree n (%)	Not applicable n (%)
*I participate in the development of the profession* (n = 303)		11 (3.6)	48 (15.8)	**119 (39.3)**	103 (34.0)	21 (6.9)	1 (0.30)
		Never n (%)	Rarely n (%)	Occasionally n (%)	Often n (%)	Very Often n (%)	Everyday n (%)
*I participate in professional discussions on professional forums, sent by email or social network* (n = 302)		44 (14.5)	**123 (40.6)**	114 (37.6)	0 (0)	14 (4.6)	7 (2.3)
*During the last year, I implemented some actions to promote the profession* (*n* = 302)		60 (19.8)	95 (31.4)	**104 (34.3)**	28 (9.2)	13 (4.3)	2 (0.70)

#### Objective 2: individual and organizational factors perceived as influencing competencies

When exploring associations from phase 1 results with perceived competency level, no significant associations were found with any of the seven roles and working in private versus public settings or working with a clientele in physical health versus mental health. However, competency level was significantly higher for time since graduation for the roles of expert in enabling occupation (*p* = 0.02) and for the change agent role (*p* value = 0.003). Competency level was also rated higher for those who supervised trainee/interns as compared to those who did not for multiple roles (see Table 4): expert in enabling occupation (*p* < 0.001), communicator role (*p* = 0.001), collaborator role (p = 0.02), change agent role (*p* = 0.04) and scholar role (*p* = 0.03).

**Table 4:** Exploration of associations using the Chi square test (or Fisher’s exact test) between perceived competency level for the seven roles with time since graduation and supervision of trainee/interns

Perceived competency level	Time since graduation		Preceptor role	
	Less than one year n (%)	One year and more n (%)	Yes n (%)	No n (%)
**Expert in Enabling Occupation** (N = 304)				
Very high	5 (8.6)	34 (13.8)	23 (21.9)	16 (8.0)
High	12 (20.7)	91 (37.0)	43 (41.0)	60 (30.2)
Adequate	37(63.8)	115 (46.7)	38 (36.2)	114 (57.3)
Borderline	4 (6.9)	5 (2.0)	1 (0.9)	8 (4.0)
Inadequate	0	1 (0.5)	0	1 (0.5)
I don’t know	0	0	0	0
	***p*value = 0.02**		***p*value < 0.001**	
**Communicator** (N = 303)				
Very high	5 (8.6)	44 (18.0)	29 (27.6)	20 (10.1)
High	25 (43.1)	99 (40.4)	40 (38.1	84 (42.4)
Adequate	26 (44.8)	95 (38.8)	35 (33.3)	86 (43.4)
Borderline	2 (3.5)	7 (2.8)	1 (1.0)	8 (4.1)
Inadequate	0	0	0	0
I don’t know	0	0	0	0
	*p* value = 0.33^*^		***p*value = 0.001**	
**Collaborator** (N = 303)				
Very high	10 (17.2)	62 (25.3)	35 (33.3)	37 (18.7)
High	35 (60.3)	126 (51.4)	50 (47.6)	111 (56.1)
Adequate	12 (20.7)	51 (20.8)	19 (18.1)	44 (22.2)
Borderline	1 (1.8)	4 (1.6)	0	5 (2.5)
Inadequate	0	0	0	0
I don’t know	0	2 (0.9)	1 (1.0)	1 (0.5)
	*p* value = 0.67^*^		*p*value = 0.02^*^	
**Practice Manager** (N = 304)				
Very high	7 (12.1)	22 (8.9)	15 (14.3)	14 (7.0)
High	22 (37.9)	79 (32.1)	35 (33.3)	66 (33.2)
Adequate	25 (43.1)	117 (47.6)	46 (43.8)	96 (48.2)
Borderline	3 (5.2)	27 (11.0)	9 (8.6)	21 (10.6)
Inadequate	0	1 (0.4)	0	1 (0.5)
I don’t know	1 (1.7)	0	0	1 (0.5)
	*p* value = 0.25^*^		*p* value = 0.37	
**Change Agent** (N = 303)				
Very high	0	18 (7.3)	7 (6.7)	11 (5.6)
High	8 (14.0)	57 (23.2)	28 (26.7)	37 (18.7)
Adequate	27 (47.4)	128 (52.0)	57 (54.3)	98 (49.5)
Borderline	20 (35.0)	40 (16.3)	11 (10.5)	49 (24.7)
Inadequate	1 (1.8)	2 (0.8)	1 (0.9)	2 (1.0)
I don’t know	1 (1.8)	1 (0.4)	1 (0.9)	1 (0.5)
	*p*value = 0.003^*^		*p*value = 0.04^*^	
**Scholarly Practitioner** (N = 303)				
Very high	2 (3.4)	11 (4.5)	6 (5.7)	7 (3.5)
High	13 (22.4)	66 (26.9)	38 (36.2)	41 (20.7)
Adequate	26 (44.8)	132 (53.9)	46 (43.8)	112 (56.6)
Borderline	17 (29.4)	33 (13.5)	14 (13.3)	36 (18.2)
Inadequate	0	2 (0.8)	1 (1.0)	1 (0.5)
I don’t know	0	1 (0.4)	0	1 (0.5)
	*p* value = 0.13^*^		*p*value = 0.03^*^	
**Professional** (N = 304)				
Very high	12 (20.7)	68 (27.6)	30 (28.6)	50 (25.1)
High	30 (51.7)	119 (48.4)	56 (53.3)	93 (46.7)
Adequate	16 (27.6)	58 (23.6)	19 (18.1)	55 (27.6)
Borderline	0	1 (0.4)	0	1 (0.6)
Inadequate	0	0	0	0
I don’t know	0	0	0	0
	*p* value = 0.62^*^		*p* value = 0.23^*^	

^*^ Fisher’s exact test is reported as its *p* value differed from Chi square *p* value and more than 20% of cells have expected frequencies < 5

Qualitatively, both individual and organizational factors were perceived as influencing the enactment, maintenance and development of competencies. Individual factors included six subthemes: knowledge/skills, motivation, emotions, behavioural regulations and beliefs about consequences and capabilities. Knowledge/skills, especially having the skills to search for new knowledge *I think that when you get out of university you still have knowledge about how to go looking... That’s a lot. We haven’t covered all the clinical problems, but we’ve talked a lot about how to search to find solutions to our problems* [P6] and the introspection capacity *…in the sense that let’s assume … a situation that you’ve been caught off guard and you wonder if you’ve done things right... the individual’s ability to question themselves, or to want to improve their competencies a little* [P2] were perceived as highly influential and closely related to the second subtheme motivation *I think it’s still an interaction of all the knowledge... but it’s for sure that it still takes an intrinsic will to develop our interpersonal skills. In the sense that.... [Interviewer (I)]: We must be proactive, as we said earlier, in a way? P2. Yes, that’s right, exactly. [I]: Have a good motivation. P2. Exactly.* [P2]. Emotions, both positive and negative, emerged as an important factor perceived as influencing motivation *…the emotions I experience in my practice, either positive or negative yes it has an effect on my volition… sometimes when I have an overload... it reduces our energy resource to have this motivation and this volition there, or this commitment there to want to make things change*. [P8]. The subtheme four, behavioural regulation, was perceived to be closely related to managing those emotions *I don’t think emotions will be changed, that’s what I teach my clients! We will not change them, but our perception of the situations that are taking place, I think that is what we can change*. [P9]. The last two subthemes under individual factors were about beliefs about consequences which were perceived as rewarding *Bringing change and seeing, when you are with a client, you have felt useful and improved their quality of life... To make a difference in someone’s life*. [P12] and beliefs about capabilities *It scares me a little bit* [receiving interns]*, but everyone who received them was always positive. Yes, it takes time, but I think it’s P4 you were talking about earlier, it puts you in question a little bit. And it improves your reflective practice in relation to your daily work, why I do this*. [P3].

Organizational factors were composed of four subthemes: context/resources, social influences/team, climate/culture and stressors/pressure. The subtheme context/resources included the physical space available to work effectively as well as how resources are organised and shared: *We have a common server between occupational therapists and we have organized everything into themes, and when we have interesting articles or intervention tools, we also put evaluation templates on it, examples of analysis. We share all this, so this is a winning strategy* [P11]. The social influences/team emerged mostly as a facilitator where a supportive environment was said to foster positive emotions and self-confidence, *I think it’s really the team, the peers. I’d put that as a priority. It is really the people who will work with you who will support you or not, depending on the dynamics of the team*. [P14]. *The team, I think that if you’re in a good working environment, that’s really key…It also promotes self-confidence*. [P13]. The climate/culture of the organization also emerged as an influential factor *I was in a practice environment that was open, and they were very important, very valued. But if I were in an environment that is very routine, I don’t think it would have allowed me to develop that skill, if for them change was synonymous, I don’t know, with wasting money.... Now it would have stopped it, it would have been an obstacle*. [P1]. The last subtheme was stressors/pressure from the working environment mainly due to heavy workloads: *There was a period when it was really “survival mode”. I had to answer the queries, but I didn’t see myself going any further, developing tools, dwelling on my practice. I didn’t have time to do that. So that’s really what I think the main obstacle is, the lack of time related to the ever-increasing workloads.* [P1].

Individual factors were perceived as easier to modify as indicated in this quote: *I think that in an intrinsic way there are still many that can be modified, such as interpersonal skills, abilities, knowledge, professional identity, our emotions. It’s something I think it’s modifiable, voluntarily. In the sense that we can modify it, we can learn from all of this and we can refine our practice*. [P8] as compared to organizational factors *I think there are some extrinsic factors that are more difficult to change... It doesn’t mean it can’t be changed, but it may take longer. Then to look for other resources, to be able to make these changes if we think it is for the best. So it’s not to say that external factors are not changeable, but it’s certainly sometimes it can be more difficult* [P15].

#### Objective 3: strategies used to maintain and keep developing competency level

The scholar role was especially targeted as one needing further development and as a foundation for the expert role. It included critical/reflexive practice, the use of evidence and peers/colleagues for support as well as a source of evidence: *Yes, it’s easier* [ask a colleague instead of searching evidence]*. We know we’re going to get a quick answer. While evidence takes longer to find the answer to the questions we are asking ourselves* [P11]. Continuing education and supervision of interns which was also mentioned as a strategy to foster critical/reflexive practices were viewed as sources of evidence: *Yes, I had two interns and I will have more this year and yes, I agree that it helps a lot. Because it allows us to think a little bit about what we do, because we have no choice, because we have to explain why we do what we do. And also the students also bring us information that we don’t necessarily have the time to look for, so yes I find that it allows us to develop the role of expert* [P7]. The strategies reported in phase 1 as being most used to promote reflective practice included informally talking to colleagues about difficulties encountered in practice (273/303; 90.1%), analyzing one’s own critical incidents encountered in practice (145/303; 47.9%) followed by discussion groups about practice (136/303; 44.9%) and mentor support (122/303; 40.3%).

## Discussion

### Enactment of competencies

The study sought to explore how competencies [4] of occupational therapists with an entry-to-practice master’s degree perceived their ability to enact competencies in clinical practice. Though the response rate was 26%, which could be seen as a limitation, this is considered an acceptable response particularly given the debates around the representativeness of the sample as being more important than actual number of respondents [34]. However, we acknowledge that we do not have relevant information about non-participants [35] despite being highly confident regarding the denominator used to calculate our response rate. Specifically, we aimed to describe clinicians’ perceived competency level, the individual and organizational factors perceived as impacting the enactment of competencies and to describe the strategies used to maintain and further develop their competency level for the seven roles. It is reassuring to find that the vast majority (96.7%) of respondents perceived their competency level to be adequate or higher for the central role of expert in enabling occupation. Equally reassuring is the finding that the competence level for this role, along with the change agent, are perceived as high or very high for those with more years of clinical practice experience. This finding is aligned with the definition of a competency-based approach at initial training where experience (i.e. training in real life, authentic situations) facilitates both knowledge acquisition/consolidation and more efficient knowledge mobilization [7, 36] as well as the theory of lifelong learning [37]. However, the change agent role (equivalent to health advocate role in CanMEDS) [5], which in part includes advocating for occupation [4], was perceived below the minimum level required to practise (i.e. below an ‘adequate’ rating) for one fifth of respondents. It was presumably less requested in large part due to its focus on more challenging and emerging areas of practice in the profession [38], although one does not need to be in a management position to enact this role. In contrast, the collaborator and communicator roles were perceived with a higher competence level as compared to the other roles. Both roles were considered highly valued by the practice environment and highly solicited on a daily basis. Indeed, occupational therapists collaborate with several different professionals and we wonder if this is the case for other professions, or if this is an inherent characteristic of the occupational therapy profession because of their holistic scope of intervention [30]. Focus group participants confirmed the close interrelation of those two roles; collaborating with several other professionals affords with a multitude of opportunities to enact their communicator role. They also asserted that documentation was the most time consuming after direct time to clients; they perceived this as a challenge and reported a desire to decrease the time devoted to writing.

The scholar role clearly mentioned as a foundation for the central role of expert and was targeted as THE role to maintain and keep developing in order to remain at an expected level of expertise; nonetheless 17.2% of respondents perceived their competence to be less than adequate for this role. Scholarly practice involves both reflective practice and an evidence-based approach to care which we know has been a challenge for the profession for many years [39]. Focus group participants mentioned having to look for evidence on their own time which requires lots of motivation and is often challenging with the professional and personal demands on clinicians [40]. As evidence-based practice and reflective practice have become important key competencies [4] and increasingly valued by most organizations, we anticipate (and look forward to) a greater recognition of time devoted to the scholar role in practice in the future to engage in knowledge translation and continued professional development activities.

### Factors perceived to influence competency

When looking at the factors perceived as influencing competency enactment, maintenance and development, we were not surprised to find as many intrinsic (individual) as extrinsic (organization/contextual) factors. Indeed, given that the acquisition and expression of competencies depend on both the characteristics of the professional and their workplace environment, this finding mirrors the growing body of literature in the interaction of these two areas [41, 42]. Our data preclude us from discussing the potential influence of gender or age as our sample is representative of the targeted population and is predominantly female (95%) and young (28.4 ± 3.9). The characteristics of the work team and the organization can influence how competencies are manifested in practice [43]. In fact, we found that team climate/culture and perceived stressors/pressure (especially relating to a heavy workload) trigger emotions (e.g. pleasure, frustration or anger), which may positively or negatively influence one owns beliefs about capabilities and consequences. Though the interaction between all those factors is complex and not yet clearly substantiated through empirical research, many of these were perceived as potentially modifiable; indeed, the individual factors are more amenable to change as clinicians believe that they have more control over these (as compared to organizational/contextual factors).

### Strategies used to maintain and develop competencies

Strategies used in practice to maintain and develop further competencies mostly targeted the scholarly practitioner role (i.e. evidence and reflective practice) with the ultimate aim of improving clinical expertise [42, 44]. Strategies such as continuing professional development activities, supervision of students and the use of peers/colleagues by way of a formal or informal mentor or an OT Facebook page were mentioned as a source of evidence. Being a preceptor was found as an effective strategy for skills’ development [45] and a positive association was found in our results between a higher perceived competence level and five of seven roles (expert, communicator, collaborator, change agent and scholar). However, we may also hypothesise that those who perceived themselves at a higher level of competence are more prone to take on the preceptor role. Another major strategy mentioned in our results and supported by the literature [46] was the use of peers. Indeed, a recent scoping review on competence assessment and maintenance [1] found that informal consultation with peers was favoured over formal learning and the working environment was found as having a huge influence on competence maintenance and development. It may be that the integration of knowledge in practice is only possible within a social learning context, that is, a context that allows for networking, feedback and reflection supported by the culture of the workplace [1, 47]. Indeed, using peers who have the knowledge was used as a first step rather than looking up by oneself research evidence; tacit knowledge being essential for good judgment in the perspective of integrating evidence, expertise and values [48].

### Strengths and limitations

The two-phase mixed methods design consisted of the quantitative results of phase 1 which were then further discussed through focus groups (in phase 2) is a strength of this study [24]. The sample size could be considered as another strength although clear cut-offs of what an acceptable response rate would be for surveys remain a controversial topic [34]. An important limitation, however, is the varying missing data throughout phase 1 (survey). We also acknowledge that using self-report is a limitation and thus results must be interpreted with caution. Finally, associations between perceived competency level and variables such as time since graduation or being in a preceptor role were considered exploratory and as such should also be interpreted with caution since a multivariate approach would be required to control for confounding factors.

## Conclusion

This two-phase sequential mixed methods study provides valuable information as to how the seven roles are enacted in practice. The seven roles are not equal hierarchically neither are they valued equally by clinicians and organizations. An important but surprising challenge that remains is relative to the scholar role which is fundamental for maintenance and development of the expert in enabling occupation role. Despite being described as valued by clinicians and organizations, insufficient time is allotted to searching for evidence and reflection in practice. This appears to force clinicians to enact their scholar role in their personal time and creates a dichotomy between the scholar being a role that is inherent to practise versus outside of practice. This study highlights multiple factors, both individual and organizational, perceived as influencing competence enactment, maintenance and development and the complex interactions within those factors. Strategies emerging from the results are mainly directed toward the scholar role and an emphasis stood out on the use of peers as a source of evidence. Future studies should look at how contextual factors influences the enactment of competencies across different professions as well as how these evolve over time.

## Data Availability

The data used and analysed during the current study are available from the corresponding author on reasonable request.

## Abbreviations

I: Interviewer
OT: Occupational therapy
OTs: Occupational therapists
P: Participant
Profile: Profile of Occupational Therapist Practice in Canada
TDF: Theoretical Domains Framework
